# Behavioral and Brain Structural Changes in Kindled Rats Induced by Coriaria Lactone/Pentylenetetrazol

**DOI:** 10.3389/fnbeh.2021.727872

**Published:** 2021-09-07

**Authors:** Shixu He, Xiangmiao Qiu, Jing Wang, Linghui Yang, Anjiao Peng, Wanling Li, Bosi Dong, Yusha Tang, Wanlin Lai, Lei Chen

**Affiliations:** ^1^Department of Neurology, West China Hospital, Sichuan University, Chengdu, China; ^2^Laboratory of Anesthesia and Critical Care Medicine, Translational Neuroscience Center, West China Hospital, Sichuan University, Chengdu, China

**Keywords:** epilepsy, kindling, behavior, brain structures, machine learning

## Abstract

Epilepsy is a common chronic neurological disease that is characterized by spontaneous seizures. It is commonly comorbid with behavioral and mood disorders. No studies have yet examined the behavioral or structural brain changes associated with coriaria lactone (CL)-induced and pentylenetetrazol (PTZ)-induced kindlings. This study examined whether the increased seizure susceptibility induced by CL/PTZ is accompanied by behavioral impairments and aimed to identify associated structural brain changes. Kindling models were induced using CL and PTZ, with 10 rats in each group. After successful kindling, rats were subjected to brain structural imaging using T2-weighted imaging and underwent behavioral tests, namely, the open field test, water maze tasks, and contextual fear conditioning. Voxel-based morphometry was then used to identify possible brain structural changes associated with kindling and/or behaviors. Support-vector machine learning was also applied for the integrative analysis of behavioral changes and structural brain imaging. In the open field test, both the CL (*P* = 0.04) and PTZ groups (*P* = 0.002) spent more time in the central area than the control group. Only the PTZ group (50.29 ± 29.56 s) showed a freezing time that was significantly less than that of the control group (94.8 ± 41.04 s; *P* = 0.024, Tukey's HSD-corrected) in contextual fear conditioning, which is suggestive of impaired fear-associated learning ability. Furthermore, brain imaging analysis revealed that the gray matter volume (GMV) of the hippocampus changed in both the CL and PTZ groups when compared to control. The support-vector machine learning model indicated that the retrosplenial dysgranular and primary somatosensory cortices were associated with both of the mentioned kindling models. Furthermore, the support-vector regression model results indicated that kindling-associated GMV changes can be used to predict general exploratory activity in the open field test. In conclusion, this is the first study to report greater general exploratory activity in a CL-induced kindling model. Moreover, the general exploratory activity in the open field test can be predicted by the GMV of brain regions associated with kindling.

## Introduction

Epilepsy is a common chronic neurological disease that is characterized by spontaneous seizures. It is commonly comorbid with behavioral and mood disorders (Keezer et al., 2016; Scott et al., 2017; Hingray et al., 2019). In recent years, increasing evidence has indicated that epilepsy and behavioral and mood disorders share common underlying pathophysiological mechanisms (Chang et al., 2011; Helmstaedter and Witt, 2017; Hingray et al., 2019). However, these mechanisms underlying behavioral and mood disorder comorbidities in epilepsy remain elusive (Zarcone and Corbetta, 2017; Hoyt et al., 2018).

Kindling, which has been widely employed as an animal model of temporal lobe epilepsy, refers to the repetitive and intermittent administration of sub-convulsive stimuli to increase seizure susceptibility and progressively amplify seizure severity (Dhir, 2012). When the kindling is successful, animals develop seizures even with a sub-threshold stimulus (Dhir, 2012). Both electrical and chemical methods can be used for kindling (Kandratavicius et al., 2014). For instance, multiple chemical agents, including pentylenetetrazol (PTZ), cocaine (Miller et al., 2000), and lidocaine (Simon et al., 1982), can accomplish chemical kindling and subsequently induce behavioral seizures.

Given that different convulsant agents have different mechanisms of action, kindling models induced by various agents might show inconsistent behavioral and brain structural changes, which could help us understand the relationship between epilepsy and behavioral/mood disorders. Coriaria lactone (CL) is a mixture extraction from a Chinese medical herb called *Coriaria sinica* Maxim (Wang et al., 2003). Initially used as a remedy for schizophrenia in traditional Chinese medicine, CL was eventually found to be epileptogenic when patients developed seizures after CL administration (Stables et al., 2002). However, CL was later confirmed to be a convulsant in different experimental animals, such as rodents, rabbits, and monkeys and so was then developed as a chemical epileptogenic agent for establishing epilepsy models, including acute seizure models, kindling models, and status epilepticus models (Wang et al., 2003; Hong et al., 2013; Cheng et al., 2015). To date, CL has been repeatedly used for the construction of kindling models, with satisfactory mortality and kindling rates being reported (Hong et al., 2013). The mechanisms of CL-induced kindling have also been reported to be associated with increased intracellular calcium and the subsequent activation of calcium-dependent signaling pathways (Zhang et al., 2009), which differ from PTZ-induced kindling. However, no studies have yet examined the behavioral and structural brain changes associated with CL-induced kindling. It, therefore, remains unknown whether CL-induced kindling models can develop behavioral and mood impairments. The goal of the study was to examine whether increased seizure susceptibility induced by CL or PTZ is accompanied by behavioral symptoms and to identify the associated structural brain changes.

## Materials and Methods

### Animal Model Construction

We constructed two kindling models. In this study, the widely used PTZ-induced kindling model was used as a comparison model. The construction of the CL-induced kindling model followed the same animal model process as PTZ-induced kindling, which administers the chemical agents every other day until kindling is successful. A control group was also included in this study. Each group was randomly allocated to three groups, with 10 male Wistar rats per group (weight 275–315 g, Chengdu Dossy Experimental Animal Co., Ltd., Chengdu, China). Rats had *ad libitum* access to food and water and were group-housed with five rats per cage. A 12-h dark–light cycle (8:00 p.m. to 8:00 a.m.) was set. Room temperature was kept from 20 to 26°C, and relative humidity ranged from 40 to 70%. A picric acid solution was used to mark the animals.

A subthreshold dose of PTZ (30 mg/kg, P6500, Sigma-Aldrich, St. Louis, MO, USA) and CL (1.75 mg/kg) dissolved in 0.9% NaCl was intraperitoneally administrated to the two groups, respectively. A previous study (Wang et al., 2003) already reported 1.75 mg/kg as the subthreshold dosage with the most satisfying kindled rate in the SD rat strain. Thus, we chose 1.75 mg/kg and verified it as a subthreshold in our preliminary experiment using Wistar rats. Animals were observed for 30 min after each injection to rate seizures according to the Racine scale scoring system (Racine et al., 1973), which is a widely used scoring system for seizure severity. In this scale, higher scores represent more severe behavioral expression, specifically, stage 1: immobility, mouth and facial movement including eye closure, twitching of the vibrissae, sniffing, and facial clonus; stage 2: head nodding, which indicates more severe facial clonus; stage 3: forelimb clonus; stage 4: rearing, often accompanied by bilateral forelimb clonus; and stage 5: all of the previous stages plus a loss of postural control, accompanied by generalized clonic seizures. Rats were considered to have been kindled if three consecutive stage 4 or stage 5 seizures occurred according to the kindling model construction protocol from Dhir (Dhir, 2012). After that, the subthreshold administration of the two chemical convulsants was terminated. The same amount of saline was then given to the control group at the same frequency. Two rats in the PTZ group, one rat in the CL group, and one rat in the control group died during the injection period. This study was approved by the Animal Ethics Committee of West China Hospital (approval code: 2018115A). The experiments were conducted according to the national guidelines concerning animal experiments. The principles described in the Animal Research: Reporting of *in vivo* Experiments (ARRIVE) guidelines and the Basel Declaration were considered.

### MRI Acquisition

Imaging was conducted 1 week after the last seizure and before the behavioral tests. A surface coil (with a diameter of 72 mm) was used during image acquisition with a 7T magnet (Bruker BioSpec 70/30 USR, Ettlingen, Germany) for all rats (*n* = 26). A coronal turbo spin-echo Rapid Acquisition with Relaxation Enhancement was performed for T2-weighted imaging. The T2-weighted imaging parameters were as follows: repetition time = 4,202 ms; echo time = 33 ms; field of view = 30 × 30 mm^2^; a 0.5-mm slice thickness; 40 slices; an in-plane resolution of 0.117 × 0.117 mm^2^ (matrix 256 × 256). A bite-bar and a gas mask were also used to keep the rats in prone positions on the MRI bed. A mixture of 2–4% isoflurane and 1.5 L/min of O_2_ was used for anesthetization. Breathing was monitored throughout the scan. For each scan, the total acquisition time was 5 min.

### Behavioral Tests

All behavioral tests were performed in the afternoon and assessed general locomotor activity, anxiety-associated behavior, spatial memory, and fear memory. Tests were carried out in the following order: the open field test, water maze task, and then contextual fear conditioning. The inter-test interval was at least 3 days. After each test, rats were returned to their original cage and given *ad libitum* access to food and water.

In the open field test, acclimated rats were individually transported into the test room and placed at the center of a square chamber (size: 90 cm × 90 cm, height of chamber walls: 40 cm). Rats were allowed 5 min to freely explore the chamber. The central area was 45 cm × 45 cm. An automated system (SMART, version 2.5.20, Barcelona, Spain, www.panlab.com) with a motor monitoring software was used to record and score exploratory activity. The total distance traveled, distance from the central region, time spent in the central region, and the number of entries into the central region were recorded.

In the water maze test, we applied a hidden platform test. Rats performed two trials every day for five consecutive days. In each trial, each rat was placed facing the pool wall in one of the four equal quadrants and then given 90 s to find the hidden platform. If successful, the rats were permitted to remain on the platform for 30 s to observe the location of the platform relative to visual-spatial cues (which were geometric images hung on the walls). If unsuccessful within 90 s, the rats were physically placed on the platform. The inter-trial interval was set at 30 s. On day 6, the hidden platform was removed, and each rat was placed in the pool and tracked using a video tracking system to measure the strength and accuracy of the memory of the previous platform location; to determine this, the total distance traveled, time and distance spent in each quadrant, percentage of time spent in the previous target quadrant, and the number of crossings over the previous platform location were recorded.

In the contextual fear conditioning, we placed each rat in the illuminated chamber for a 3-min habituation period, during which rats could explore the chamber to take in all its aspects. Rats were then given a 2-s 0.8 mA foot shock. Rats were then removed from the chamber 15 s after the shock. On the second day, rats were placed back in the same chamber for 3 min to measure freezing behavior. In both the training and test sessions, freezing time, freezing score, and freezing episodes were recorded using the ANY-maze software.

### Structural Brain Imaging Analysis

A voxel-based morphometry analysis was performed for the T2-weighted imaging analysis using the Statistical Parametric Mapping 12 (SPM12; Wellcome Trust Centre for Neuroimaging, London, UK) toolbox in MATLAB 2013b (MathWorks, Natick, MA, USA). First, the brain extraction was conducted using the Brain Extraction Tool in the FMRIB Software Library (FSL) (version 4.0) (http://www.fmrib.ox.ac.uk/fsl/). Second, the brains were aligned to a Wistar rat template (Valdes-Hernandez et al., 2011) using Linear Image Registration Tool (FLIRT) of FMRIB (Jenkinson and Smith, 2001) in FSL. Then, modulation with a 2-mm full-width at half-maximum Gaussian kernel and smoothing were conducted. After that, images were segmented into white matter, gray matter, and cerebrospinal fluid images. By summing the values of the gray matter, white matter, and cerebrospinal fluid maps, we also calculated total intracranial volume (TIV).

### Statistical Analysis

An ANOVA, applied in SPM 12, was used to generate F maps for the comparisons of gray matter volume (GMV) between the CL, PTZ, and control groups. *Post-hoc* comparisons were performed to identify brain structural changes associated with kindling and the different changes between the CL and PTZ models. Using the same template for the alignment described above, the Modified xjView toolbox (http://www.alivelearn.net/xjview) was used to visualize images.

We applied significantly different clusters (*P* < 0.10, clusters size > 50 voxels) as features by generating a mask for subsequent machine learning classification. A support-vector machine (SVM) learning method was applied for classification with a non-linear kernel function. A leave-one-out test and a permutation test with 1,000 repetitions were carried out to validate the SVM learning model, with the SVM learning being conducted using the LIBSVM toolbox (Chang and Lin, 2011) in Matlab. To identify behavioral changes associated with kindling models, we performed a one-way ANOVA and *post-hoc* analysis. Graphs were created using R (version 3.5.0).

## Results

### Kindling Model Construction

**Figure 1:**
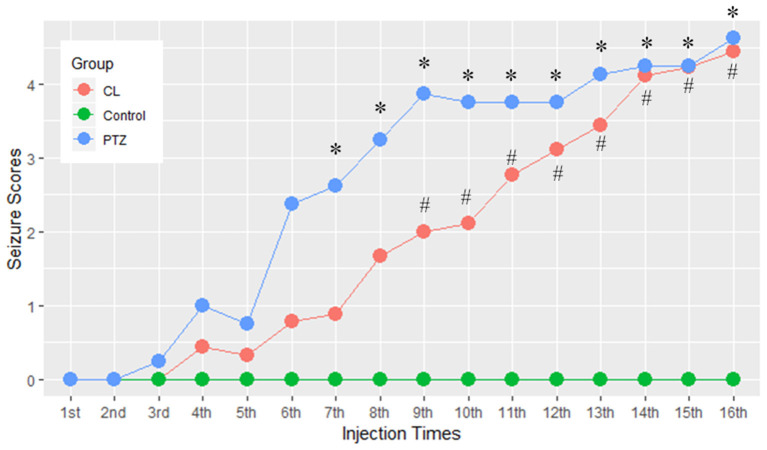
Effect of coriaria lactone (CL)/pentylenetetrazol (PTZ) on the seizure scores for 16 injections. *PTZ group showed significant larger seizure scores *vs*. control, *P* < 0.05; #CL group showed significantly larger seizure scores vs. control, *P* < 0.05.

One rat in the CL group, two in the PTZ group, and one in the control group died during the injection period. It required a maximum of 16 injections for the CL group (ranging from 13 to 16 injections) and the PTZ group (ranging from 8 to 16 injections) for the rats to be kindled successfully. Figure 1 demonstrates the seizure scores for every other day of injections. As we can see, the seizure score of the CL group did not rise as sharply as the PTZ group. From the 7th injection, the seizure score significantly differed statistically between the PTZ-induced kindling group and the control. As for the CL group compared with control, from the 9th injection onward, the seizure scores differed significantly. Eventually, all surviving rats in the two kindling groups were successfully kindled and included in the subsequent analysis.

### Identifying Behavioral Changes Associated With Kindling

**Figure 2:**
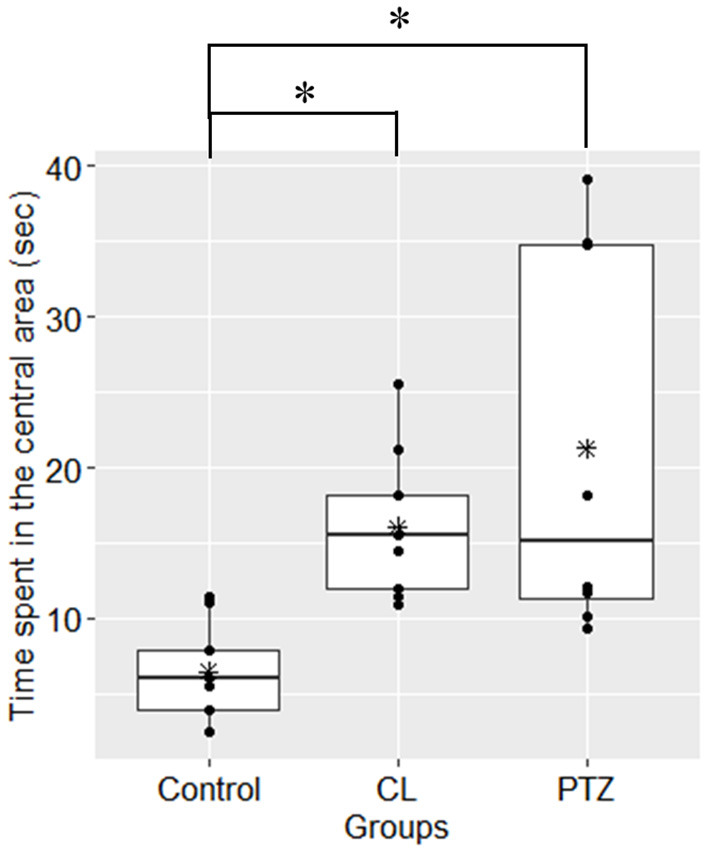
Time spent in the central area (s) in the open field tests. Both the CL and PTZ groups showed greater time spent in the central area. ^*^P < 0.05.

In the open field test, the percentage of time spent in the central area differed between groups (ANOVA, *P* = 0.002, Figure 2). Further multiple comparisons (Tukey's HSD-corrected) revealed that the PTZ group (*n* = 8, 21.26 ± 12.75 s, *P* = 0.002) and CL group (*n* = 9, 16.07 ± 4.84 s, *P* = 0.04) spent more time in the central area than the control group (*n* = 9, 6.47 ± 3.13 s). The proportion of time spent in the central area was also significantly different between groups (ANOVA, *P* = 0.002; PTZ group 7.09%, CL group 5.36%, control group 2.16%).

**Figure 3:**
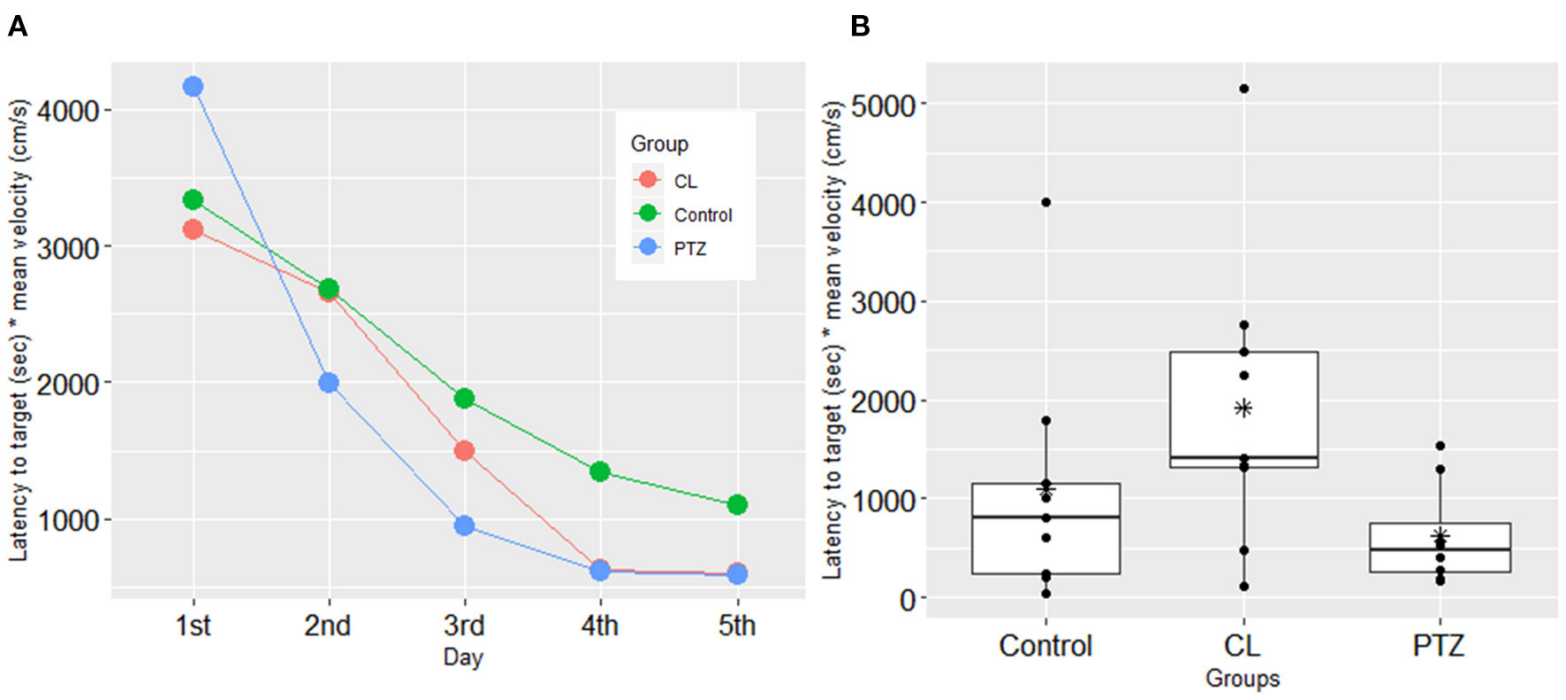
Spatial learning in the water maze test in the three groups. **(A)** In the training session, no significant difference was found in the latency to the target platform (s) multiplied by mean velocity (cm/s); **(B)** in the testing session, no significant difference was shown in the latency to the target platform (sec) multiplied by mean velocity (cm/s) in the testing session. The symbol * represents the mean of each group.

In the water maze tasks, to control for the confounding effects of velocity, we used the latency time to the platform multiplied by the mean velocity as the dependent variable for the analysis. However, no significant changes compared to control were found on the testing day (ANOVA, *P* = 0.09). During the training session, we also failed to detect significant changes in spatial learning between the three groups. Figure 3 shows the results from the training and the testing session for the three groups. Although there was a trend toward a shorter latency to the target platform in both kindling models compared to the control group, there were no significant differences.

**Figure 4:**
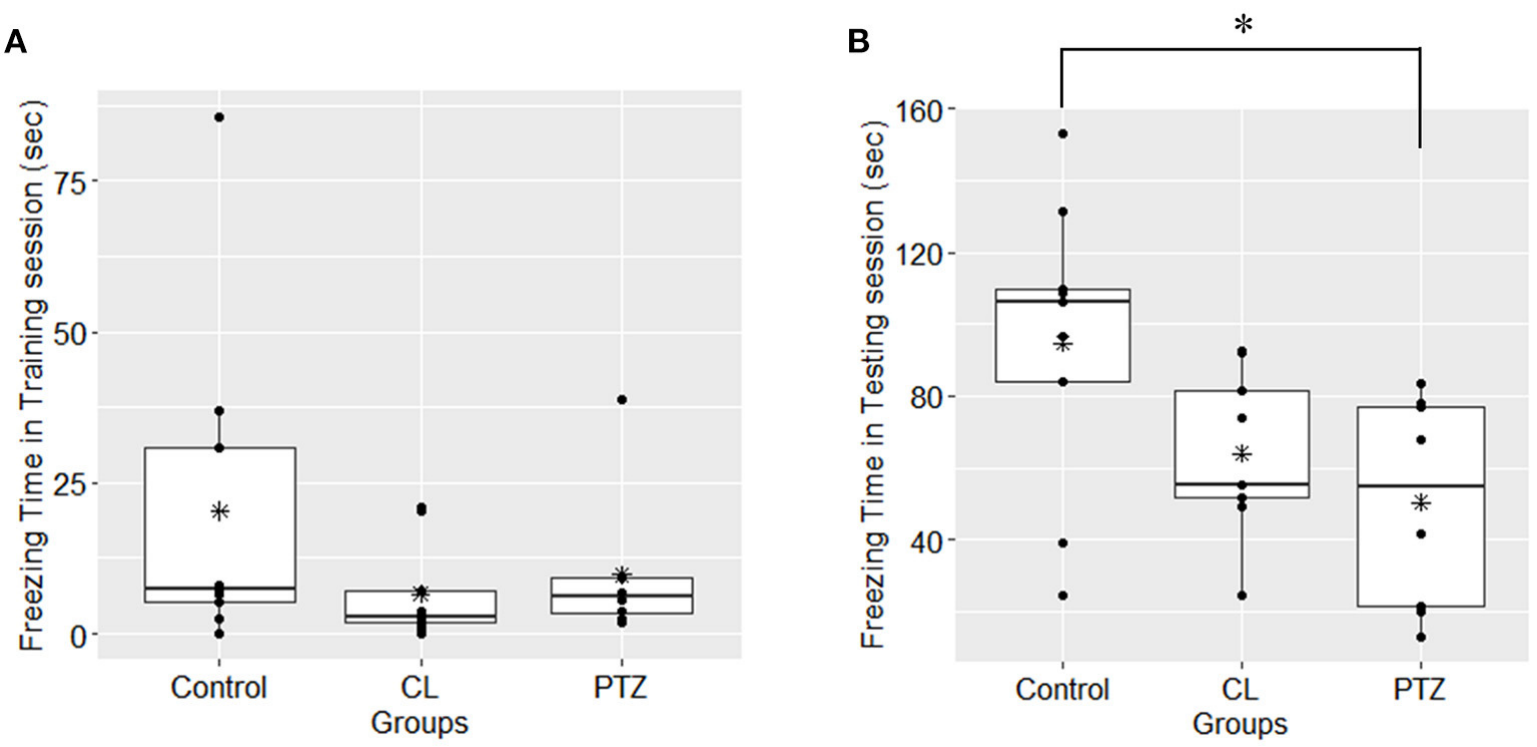
Freezing time (s) in contextual fear conditioning in the three groups. (A) In the first-day training session, no significant difference was found among groups upon the freezing time; (B); in the second-day testing session, the PTZ group demonstrated less freezing time. ^*^P < 0.05.

In the contextual fear conditioning, there were no significant differences in freezing responses on the first day of the acquisition training session, which indicates that there were no significant changes in exploratory activities (*P* = 0.269, ANOVA). However, on the second day of the testing session, an ANOVA revealed that there were between-group differences in freezing time (*P* = 0.025, ANOVA, Figure 4), with the PTZ group (*n* = 8, 50.29 ± 29.56 s) demonstrating a significantly shorter freezing time (*P* = 0.0235, Tukey's HSD-corrected) than the control group (*n* = 9, 94.8 ± 41.04 s), which indicates worse associative learning and impaired fear memory. No significant difference in freezing time (*P* = 0.126, Tukey's HSD-corrected) was found between the CL group (*n* = 9, 63.98 ± 22.67 s) and the control group.

### Identifying Brain Structural Changes Associated With the Two Kindling Models

We first evaluated the impact of kindling on voxel-based structural brain changes using GMV maps. We calculated the TIV of each rat and found significant between-group differences (Kruskal–Wallis rank-sum test, *P* = 0.012); however, a *post-hoc* analysis revealed no differences between the CL/PTZ groups and the control group. As a result, we did not control for TIV in the subsequent analysis.

**Figure 5:**
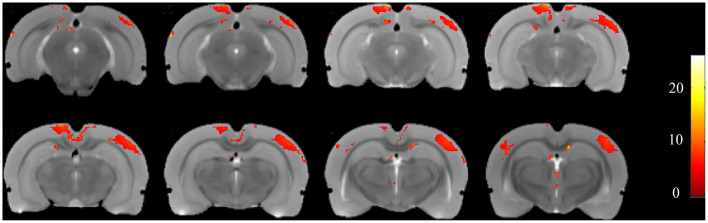
F-maps of the gray matter volume (GMV) from the ANOVA analysis of the CL, PTZ, and control groups. Only significant differences with a cluster size exceeding 50 (*P* < 0.01) are shown. At the level of the cortex, volumetric changes were found in the retrosplenial dysgranular cortex and primary somatosensory cortex.

Using voxel-based morphometry, F-maps from the ANOVA analysis were generated, as shown in Figure 5. T-maps between each pair of comparisons were also generated to examine GMV changes. The results are summarized in Table 1. Compared with the control group, 19 clusters demonstrated GMV changes when setting the *P*-value at 0.005 and the minimum cluster size at 50, including increases of GMV in CA1 of the right hippocampus and CA2 of the left hippocampus. For the CL group *vs*. control group, CA3 of the left hippocampus was found to have higher GMV, while CA1 of the left hippocampus had a decreased GMV. For the CL group *vs*. PTZ group, the decreased GMV of multiple brain regions was also found, as shown in Table 1. Among these brain regions was the bilateral dorsal intermediate entorhinal cortex.

**Table 1:** Changes in gray matter volume (GMV) as compared to the control group.

	**Volume change**	**Region**	**Maximal T values**	**Total voxels**
PTZ-con	Increase	V1, V1M, V2L, V1B	4.8592	1,765
	Increase	RSD (left + right)	4.5444	492
	Increase	Au1_left	5.7312	141
	Increase	CA1_right	4.3731	71
	Increase	CA2_left	4.2635	204
	Decrease	Prelimbic cortex_left	−5.132	326
CL-con	Increase	CA3_left	4.0514	54
	Decrease	RSD_right	−4.4347	44
	Decrease	S1ULp_left	−3.9735	131
	Decrease	CA1_left	−3.5087	52
CL-PTZ	Decrease	V1B_left	−3.5026	50
	Decrease	RSD_right	−5.5053	86
	Decrease	Au1_left	−6.717	242
	Decrease	DIEnt_right	−4.9762	60
	Decrease	DIEnt_left	−4.3802	65

*V1M, primary visual cortex, monocular area; V1B, primary visual cortex, binocular area; V2L, secondary visual cortex, lateral area; RSD, retrosplenial dysgranular cortex; Au1, primary auditory cortex; S1ULp, primary somatosensory cortex, upper lip region; DLEnt, dorsolateral entorhinal cortex; LptA, lateral parietal association cortex; AuV, secondary auditory cortex, ventral area; Ect, ectorhinal cortex; DIEnt, dorsal intermediate entorhinal cortex*.

**Figure 6:**
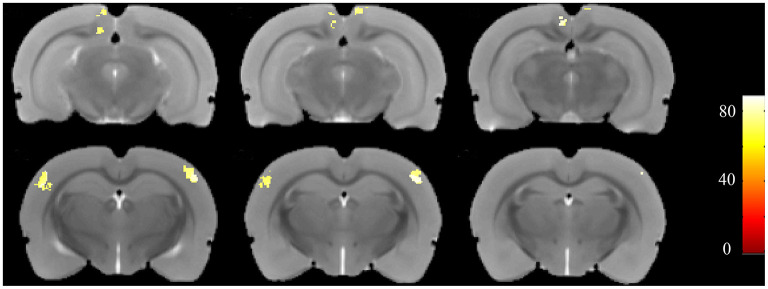
Accuracy maps from the support-vector machine (SVM) based on GMV maps. Only meaningful clusters with accuracies >70% and a cluster size exceeding 50 are shown.

Furthermore, to identify possible brain structural changes associated with kindling, we pooled the CL and PTZ groups together as one group and used SVM to classify between kindling and control. The overall accuracy for the use of GMV for discrimination was 88.89%. As shown in Figure 6, Table 2, the GMV of the retrosplenial dysgranular cortex and primary somatosensory cortex had a significant discriminative effect.

**Table 2:** The top regions that contributed to kindling-control classification (SVM) based on GMV maps.

**Region**	**Number of voxels**	**Peak accuracy**	**Peak –log** ***P***
RSGb_left	101	85%	3.00
RSD_left	660	85%	3.00
RSD_right	302	81%	3.00
S1BF_left	381	85%	3.00
S1BF_right	1,022	89%	3.00

*RSD, retrosplenial dysgranular cortex; S1BF, primary somatosensory cortex; RSGb, retrosplenial granular cortex, b region*.

### Possible Associations Between Behavioral Changes and Structural Brain Changes

To identify the possible associations between behavioral indices and structural brain changes, we used support-vector regression analysis. First, we evaluated whether GMV could be used to predict behavioral changes in kindling models. For the GMV maps, we first set all the brain regions as independent variables, while dependent variables were set as the behavioral indices, including time spent in the central area for the open field test and freezing time in the contextual fear conditioning; with this analysis, we failed to find a significant result concerning the association between behavioral indices and structural brain changes. We then entered brain clusters that showed the different GMV between groups in the previous ANOVA results as independent variables; the non-linear regression model showed a significant association between the predicted and actual times spent in the central area (*r* = −0.69, *P*-value from the permutation test: 0.016). This result suggested that these brain regions might contribute to performances in the open field test, but with a non-linear pattern.

## Discussion

The main findings of this study were as follows: (1) the CL- and PTZ-induced kindling models demonstrated greater general exploratory activity, as in contextual fear conditioning, only the PTZ-induced kindling model showed impaired performance, suggesting impaired contextual associative learning; (2) machine learning models revealed that the retrosplenial dysgranular cortex and primary somatosensory cortex were associated with the two kindling models; (3) the support-vector regression model results suggested that brain regions associated with kindling can be used to predict general exploratory activity.

In recent years, evidence from both clinical and animal studies has indicated that there may be common underlying pathophysiological mechanisms for seizure susceptibility and behavioral/mood disorders (Chang et al., 2011; Hesdorffer et al., 2012). For example, there was a previous amygdala kindling model study that demonstrated decreased motor exploratory activity after kindling (Chen et al., 2016), suggesting comorbidity with anxiety-related behaviors. However, inconsistent with the previous study, our study demonstrated increased motor exploratory activity induced by kindling, which is seemingly opposed to the hypothesis of common underlying mechanisms. Nonetheless, it has to be noted that the interpretation of increased activity in the central areas as anxiety-related performance should be carried out carefully as it could also be regarded as an adaptive exploratory response. Moreover, there have been other studies showing results consistent with ours. A seizure-susceptible strain has been reported to have increased activity in the central area in an open field test (Langberg et al., 2016). This study also used a kindling-resistant strain, which showed decreased activity across the central area. Thus, further studies are needed to fully characterize the performance of the CL-induced kindling model in open field tests.

For brain structural changes, to our knowledge, our study is the first to examine CL-induced structural brain changes. Our structural imaging results showed that the GMV of the left hippocampus (mainly in CA1 and CA3) changed as a result of CL administration, which is consistent with a previous finding that the CL-induced kindling model represents an animal model of temporal lobe epilepsy. For PTZ-induced kindling, although a previous study has shown increased T2 in the hippocampus and entorhinal cortex (Fang and Lei, 2010), our study is the first to analyze gray matter changes using voxel-based morphometry. Our results demonstrated changes in the GMV in various brain regions, including the hippocampus and visual cortex.

Other than the hippocampus, from a whole-brain perspective, pooling the CL and PTZ groups as one group revealed the involvement of the retrosplenial cortex and somatosensory cortex, which have long been recognized as key regions of the cognitive network, in kindling models. In human epilepsy studies, changes in GMV have been identified in both temporal, extra-temporal, cortical, and subcortical regions, with the hippocampus, thalamus, and parietal lobes being the preferential sites of such changes (Keller and Roberts, 2008). Compared with human studies, voxel-based morphometry studies of animal models are relatively rare. To our knowledge, this is the first study to use machine learning methods to differentiate between kindling models and control animals. The function of the retrosplenial cortex is to integrate information in the prefrontal, parietal, and occipital cortices with subcortical sites such as the hippocampus (Vann et al., 2009). There are also GABAergic neurons that project from CA1 and the subiculum to the retrosplenial cortex, which might underpin the synchronized oscillatory activity between these regions. Moreover, the number of neurons in the retrosplenial cortex has been shown to decrease in electrical kindling models (Cardoso et al., 2008). However, it is unknown whether neuron projections between CA1 and the retrosplenial cortex play a role in the common underlying mechanisms of epilepsy and behavioral disorders.

We also demonstrated impaired contextual fear conditioning in the PTZ-induced kindling model, which is consistent with previous results. However, we found no such impairment in the CL-induced kindling model. Given that we did identify a trend toward decreased freezing times in the CL group, it is possible that this finding could become significant with larger sample size. Compared with PTZ, which requires a larger sample size to reach statistical significance, CL might lead to a milder impairment of contextual fear conditioning. Still, differences in the brain structures between the PTZ and CL groups might provide hints about which brain regions are associated with contextual fear conditioning. Given that the PTZ group demonstrated both increased seizure susceptibility and the impairment of contextual fear conditioning compared to the control group, the altered brain structures identified using a direct comparison between the PTZ and control groups might not only include those associated with increased seizure threshold, but also those associated with impairments in contextual fear conditioning. Conversely, the CL group only showed increased seizure susceptibility, without the impairment of contextual fear conditioning. Thus, the overlap between the PTZ *vs*. control group and PTZ *vs*. CL group comparisons might reveal the brain regions associated with contextual fear conditioning. In our study, these regions included the dorsal intermediate entorhinal cortex, primary visual cortex, and primary auditory cortex.

Using a support-vector regression model, we also found that brain regions associated with kindling could be used to predict performance in an open field test. Although the linear support-vector regression model failed to yield significant results, the significant results from the non-linear model indicated that there is a relationship between the GMV of the mentioned brain regions and performance in an open field test. However, the specific relationships between these brain regions and performances in open field tests remain unknown.

There are some limitations to this study. First, larger sample size is needed to confirm the negative results of spatial learning and recall ability and contextual fear conditioning in the CL-induced kindling model. Our study is the first to demonstrate a CL-induced changes of behavior and no previous study has reported the effect size toward every behavioral test. Thus, our estimation of the sample size was based on the general sample size required for kindling model construction and behavioral tests. However, our study results could serve as a prior study for future studies to calculate sample size more accurately. Second, the roles of the retrosplenial cortex in kindling models still need to be clarified in further research. Further investigations are also needed to determine the specific relationship between the brain regions associated with kindling and performance in the open field test. Third, we did not perform any intervention of GMV; thus, a conclusion about whether hippocampal structural changes are a primary cause or a consequence of epilepsy could not be reached. This also needs further studies to clarify.

In conclusion, our study not only demonstrated the presence of anxiety-related behaviors in a CL-induced kindling model for the first time but also found that the GMV of the retrosplenial dysgranular cortex and primary somatosensory cortex was associated with kindling. Moreover, through the integrative analysis of GMV and behavioral indices using a support-vector regression model, our study showed that GMV changes resulting from kindling might also contribute to the general exploratory activity.

## Data Availability Statement

The raw data supporting the conclusions of this article will be made available by the authors, without undue reservation.

## Ethics Statement

The animal study was reviewed and approved by Animal Ethics Committee of West China Hospital (approval code: 2018115A).

## Author Contributions

SH, XQ, and LC: conceptualization and investigation, writing original draft preparation, review, and editing, and supervision. SH, XQ, JW, and LY: methodology. SH, XQ, JW, LY, AP, and WLi: animal models. SH, XQ, JW, LY, AP, BD, YT, WLa, and LC: brain imaging and data analysis. SH, XQ, JW, LY, AP, WLi, and LC: fear conditioning. All authors contributed to the article and approved the submitted version.

## Funding

This study was supported by the National Natural Science Foundation of China (grant number: 81871018).

## Conflict of Interest

The authors declare that the research was conducted in the absence of any commercial or financial relationships that could be construed as a potential conflict of interest.

## Publisher's Note

All claims expressed in this article are solely those of the authors and do not necessarily represent those of their affiliated organizations, or those of the publisher, the editors and the reviewers. Any product that may be evaluated in this article, or claim that may be made by its manufacturer, is not guaranteed or endorsed by the publisher.
