# Supplementary material for: Behavioral and Brain Structural Changes in Kindled Rats Induced by Coriaria Lactone/Pentylenetetrazol
Source: Front Behav Neurosci. 2021 Sep 7;15:727872. doi: 10.3389/fnbeh.2021.727872 (PMC8452916; doi:10.3389/fnbeh.2021.727872)
Supplement: Supplementary file 1 [file Table_1.DOCX]

Table 1. Core Animal Characteristics Case Report Form

| **CDE Name** | **Data Collected** | | **Files Uploaded** |
| --- | --- | --- | --- |
| ***Core animal information*** | | | |
| Animal tag, mark, or other identification  If yes, specify | ☐ Yes; ☐ No; ☐ Unknown  _ picric acid solution;______ |  | |
| Copy of the cage card(s) | ☐ Yes; ☐ No | [See CRF for uploaded files to upload the copy of the cage card] | |
| Date of birth: is it known?  If the date of birth is known, specify  Note: If exact date is not known, but the week is known, enter Monday of that week | ☐ Yes; ☐ No  __/__/____ (MM/DD/YYYY) |  | |
| Weight on arrival to facility (g) | 275–315 g |  | |
| ***Animal source*** | | | |
| Vendor | Chengdu Dossy Experimental Animal Co., Ltd | |  |
| City, State (or province or county), Country | Chengdu, Sichuan , China | |  |
| Other source if there is no vendor (e.g., investigator)  If there was another source, specify Name  Institution | ☐ Yes; ☐ No; ☐ Unknown  _______  _______ | |  |
|  |  | |  |
| Breeding scheme | ☐ Yes; ☐ No | | [See CRF for uploaded files to upload information] |
| If animals were shipped, is the arrival date to the facility known?  If yes, specify date | ☐ Yes; ☐ No  __/__/____ (MM/DD/YYYY) | |  |
| Period of quarantine after arrival  If there was a period of quarantine, specify start  end | ☐ Yes; ☐ No; ☐ Unknown  __/__/____ (MM/DD/YYYY)  __/__/____ (MM/DD/YYYY) | |  |
| ***Sex and related information*** | | | |
| Males: |  | |  |
| Housed alone prior to use? | ☐ Yes; ☐ No; ☐ Unknown | |  |
| If no, specify # males/cage | 5 males/cage | |  |
| Was there fighting? | ☐ Yes; ☐ No; ☐ Unknown | | [If yes, uploaded details in the CRF for uploaded files] |
| Was animal  gonadectomized  sham-gonadectomized? | ☐ Yes; ☐ No; ☐ Unknown  ☐ Yes; ☐ No; ☐ Unknown | | [See CRF for uploaded files to upload surgical information] |
| Were there treatments to replace gonadal hormones (e.g. Testosterone)? | ☐ Yes; ☐ No; ☐ Unknown | | [See CRF for uploaded files to upload information about treatments] |
| Females: | No females were used in our study | |  |
| Housed alone prior to use? | ☐ Yes; ☐ No; ☐ Unknown | |  |
| If no, specify # of females/cage | ________ | |  |
| Was the estrous cycle monitored? | ☐ Yes; ☐ No; ☐ Unknown | | [If yes, upload the estrous cycle log in the CRF for uploaded files] |
| Was animal  ovariectomized?  sham-ovariectomized? | ☐ Yes; ☐ No; ☐ Unknown  ☐ Yes; ☐ No; ☐ Unknown | | [Use the CRF for uploaded files to add information about these procedures, if conducted] |
| Was there gonadal hormone treatment (e.g. estradiol)? | ☐ Yes; ☐ No; ☐ Unknown | | [If yes, see CRF for uploaded files to add information about treatment] |
| ***Species and strain*** | | | |
| Rat strain | ☐ Wistar;  ☐ Sprague-Dawley;  ☐ Long-Evans;  ☐Other | | If other rat strain, specify  ______ |
| Mouse strain | ☐C57BL/6;  ☐ Other | | If other mouse strain, specify____ |
| Other species  strain | _______  _______ | |  |
| ***Genetic modification*** | | | |
| Genetic modification | ☐ Yes; ☐ No; ☐ Unknown | |  |
| Transgenic?  Constitutive  Deletion  Overexpression  Mutation  Other  Conditional  Inducible | ☐ Yes; ☐ No; ☐ Unknown  ☐ Yes; ☐ No; ☐ Unknown  ☐ Yes; ☐ No; ☐ Unknown  ☐ Yes; ☐ No; ☐ Unknown  ☐ Yes; ☐ No; ☐ Unknown  ☐ Yes; ☐ No; ☐ Unknown  ☐ Yes; ☐ No; ☐ Unknown  ☐ Yes; ☐ No; ☐ Unknown | | If yes, name the gene_______  If other manipulation, specify____  [If inducible, upload methods to the CRF for uploaded files] |
| Breeding Tg x WT?  If yes, is Tg the father?  Tg x Tg? | ☐ Yes; ☐ No; ☐ Unknown  ☐ Yes; ☐ No; ☐ Unknown  ☐ Yes; ☐ No; ☐ Unknown | | [See CRF for uploaded files to upload information] |
| Viral expression? | ☐ Yes; ☐ No; ☐ Unknown | | [If yes, see CRF for uploaded files to upload information] |
| Confirmation of genotype or viral expression | ☐ PCR; ☐ Western blot;  ☐ Immunohistochemistry;  ☐ Other | | [See uploaded files CRF to upload file] |
| ***Housing*** | | | |
| Pathogen free housing? | ☐ Yes; ☐ No | | [Use CRF for uploaded files to upload health report] |
| Number of animals/cage | ☐ 1; ☐ 2; ☐ 3; ☐ >3 | |  |
| Cage size | 45cm x 35cm x 20cm  length x width x height (cm) | |  |
| Cage cover  Cage material  Cage rack or holding system | ☐ Filter top ☐ Wire ☐ Other  ☐ Clear plastic ☐ Other  ☐ Controlled air ☐ Other | |  |
| Enrichment | ☐ Yes; ☐ No; ☐ Unknown | |  |
| If enrichment, the following appears | ☐ Nesting material  ☐ Chewing material  ☐ Hiding place  ☐ Other | | [Use CRF for uploaded files to upload information] |
| Bedding | ☐ Corncob; ☐Other | | [Use CRF for uploaded files to upload information if Other is checked] |
| Room temperature (range) | 20 – 26 (°C) | |  |
| Relative humidity (range) | 40 - 70 (%) | |  |
| Lights on; off (timed)  Other (e.g., natural light) | 8:00 ; 20:00 (12:00)  ☐ Yes; ☐ No; ☐ Unknown | | [If Other, use CRF for uploaded files to upload information] |
| ***Diet*** | | | |
| Vendor | Chengdu Dossy Experimental Animal Co., Ltd | | [Use CRF for uploaded files to upload information] |
| Catalog number  City, State (or province or county), Country | _______  Chengdu, Sichuan, China | |  |
| Food delivery system  Inside the cage  On the cage cover  Other | ☐  ☐  ☐ | | [If Other, use CRF for uploaded files to upload information] |
| Water  *Ad libitum*?  Filtered? | ☐ Yes ☐ No; ☐ Unknown  ☐ Yes ☐ No; ☐ Unknown | | [If Filtered, add information about filtering to the CRF for uploaded files] |
| ***Copy and paste the rows above if there were changes after a treatment, such as the following:*** | | | |
| Number animals per cage | ☐ Yes; ☐ No; ☐ Unknown | | If yes, specify______ |
| Cage type | ☐ Yes; ☐ No; ☐ Unknown | | If yes, specify______ |
| Bedding | ☐ Yes; ☐ No; ☐ Unknown | | If yes, specify______ |
| Enrichment | ☐ Yes; ☐ No; ☐ Unknown | | If yes, specify______ |
| Temperature | ☐ Yes; ☐ No; ☐ Unknown | | If yes, specify______ |
| Humidity | ☐ Yes; ☐ No; ☐ Unknown | | If yes, specify______ |
| Diet | ☐ Yes; ☐ No; ☐ Unknown | | If yes, specify______ |

**Instructions**: This form is to be filled in for one individual animal. For uploads, include as much description as is relevant and possible. For changes before or after a treatment, copy and paste rows to include the information. For example, the chow used before shipment of an animal for experimental use may be different from the chow used after arrival; the housing of animals may be >1/cage before EEG electrodes are implanted but 1/cage afterwards. This information may be important because the diet and social housing may change experimental outcomes.

**Abbreviations:** DD = date; MM = month; YYYY = year; HH = hours (0-24); MM = minutes (0-60); C = centigrade; Tg = transgenic; WT = wild type.
